# Tat–Dependent Translocation of an F_420_–Binding Protein of *Mycobacterium tuberculosis*

**DOI:** 10.1371/journal.pone.0045003

**Published:** 2012-10-22

**Authors:** Ghader Bashiri, Ellen F. Perkowski, Adrian P. Turner, Meghan E. Feltcher, Miriam Braunstein, Edward N. Baker

**Affiliations:** 1 School of Biological Sciences,Maurice Wilkins Centre for Molecular Biodiscovery, The University of Auckland, Auckland, New Zealand; 2 Department of Microbiology and Immunology, University of North Carolina School of Medicine, Chapel Hill, North Carolina United State of America; 3 Microscopy and Graphics Unit, School of Biological Sciences, The University of Auckland, Auckland, New Zealand; French National Centre for Scientific Research - Université de Toulouse, France

## Abstract

F_420_ is a unique cofactor present in a restricted range of microorganisms, including mycobacteria. It has been proposed that F_420_ has an important role in the oxidoreductive reactions of *Mycobacterium tuberculosis*, possibly associated with anaerobic survival and persistence. The protein encoded by Rv0132c has a predicted N–terminal signal sequence and is annotated as an F_420_–dependent glucose-6-phosphate dehydrogenase. Here we show that Rv0132c protein does not have the annotated activity. It does, however, co–purify with F_420_ during expression experiments in *M. smegmatis*. We also show that the Rv0132c–F_420_ complex is a substrate for the Tat pathway, which mediates translocation of the complex across the cytoplasmic membrane, where Rv0132c is anchored to the cell envelope. This is the first report of any F_420_–binding protein being a substrate for the Tat pathway and of the presence of F_420_ outside of the cytosol in any F_420_–producing microorganism. The Rv0132c protein and its Tat export sequence are essentially invariant in the *Mycobacterium tuberculosis* complex. Taken together, these results show that current understanding of F_420_ biology in mycobacteria should be expanded to include activities occurring in the extra-cytoplasmic cell envelope.

## Introduction

Tuberculosis (TB) is a devastating and contagious infectious disease. It is estimated that *Mycobacterium tuberculosis* (*Mtb*) bacilli, the causative agent of this disease, infect one–third of the world's population, while TB claims nearly two million lives a year [1]. Complications from co–infection with HIV/AIDS and the rise of multiple–drug (MDR) and extensively drug–resistant (XDR) strains of *Mtb* make TB a worldwide concern [1]. WHO estimates that there are nearly half a million new cases of MDR–TB each year; about 5% of the nine million new TB cases of all types [1]. Although some promising anti–TB drugs are in clinical trials, there have been no new drugs against TB in the last thirty years [2]. Understanding the biochemistry and physiology of active and persistent TB will help to reveal the basis of pathogenesis, making it possible to combat the disease more effectively.

**Figure 1:**
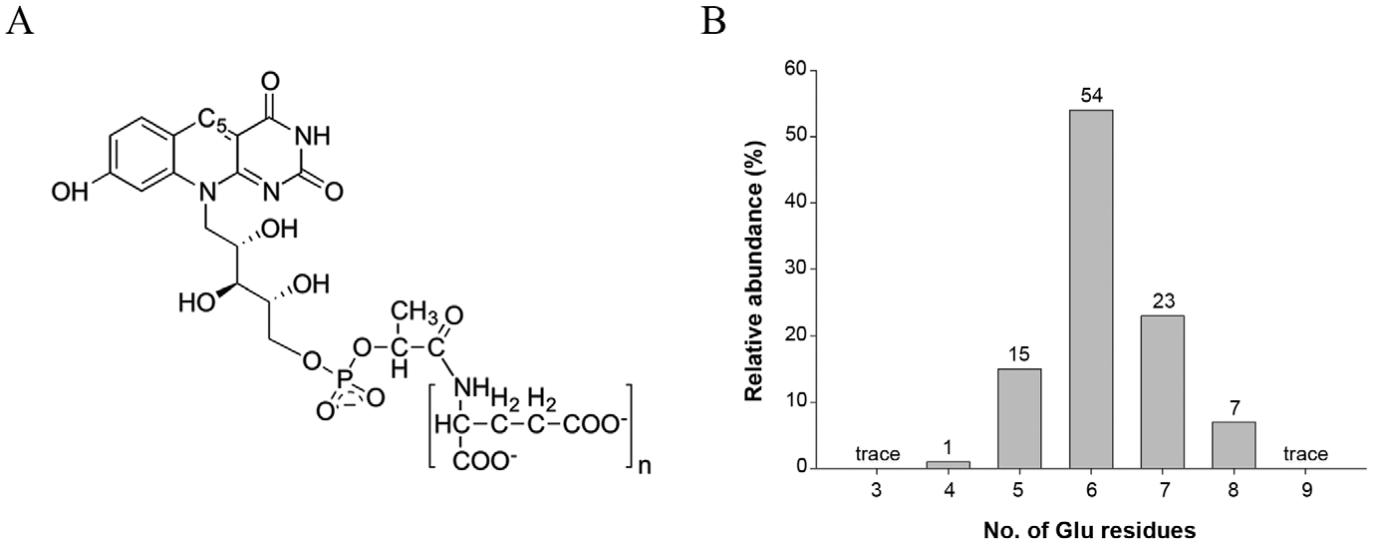
Molecular structure of cofactor F_420_. (A) Schematic representation of cofactor F_420_, where *n* varies from 2–9 in different microorganisms. (B) Mass spectrometry analysis of cofactor F_420_ bound to the purified Rv0132c–Δ38 protein showing the population of species differing in the number of glutamate residues in the poly-Glu tail.

The coenzyme F_420_ is a 5-deazaflavin derivative that has been recently proposed to play a substantial role in the redox reactions of *Mtb* [3]. F_420_ contains an isoalloxazine chromophore with a side chain composed of ribitol and phospholactate moieties and a poly–glutamate tail of variable length (Figure 1A). The isoalloxazine chromophore of F_420_ is very similar to that of the flavins (FMN and FAD), with the major difference being the atoms involved in the oxidoreductive reactions. Oxidoreduction of F_420_ is achieved by hydride transfer between a substrate molecule and C_5_ of the 5-deazaflavin moiety, whereas transfer occurs to N_5_ in FMN and FAD. Despite its structural similarity to the flavins, F_420_ is functionally similar to NAD(P)^+^, being involved in hydride transfer reactions [for a review see [4] and references therein].

The number of known F_420_–dependent proteins in mycobacterial species is growing, and a few of such activities have been experimentally shown, including F_420_–dependent glucose-6-phosphate dehydrogenase (FGD) [5], [6], deazaflavin–dependent nitroreductase (Ddn) [7], [8] and F_420_H_2_–dependent reductase (FDR) [9] activities. Bioinformatic analyses have indicated the presence of three different F_420_–dependent families in *Mtb* with at least 28 members; the luciferase–like monooxygenase (LLM), pyridoxamine 5′-phosphate oxidase (PNPOx), and the deazaflavin–dependent nitroreductase (DDN) families [3]. We have previously characterized the structure and function of FGD1 from *Mtb* and showed that it has the annotated activity, providing reduced F_420_ for cell metabolism [5]. The reaction catalysed by FGD1 is equivalent to the first step in the pentose phosphate pathway, which normally provides NADPH for reductive biosynthetic reactions and maintenance of the cellular redox state. The enzyme Ddn uses the reduced F_420_ produced by FGD1 to activate a promising anti–TB prodrug PA–824 [10], which is currently in the second phase of clinical trials. The physiological role of Ddn, however, is yet to be identified. It has also been shown that reduced F_420_ can convert NO_2_ to NO *in vitro*, implying a possible protective role against nitrosative damage in mycobacteria *in vivo* [11]. All these observations point to the fact that cofactor F_420_ has an important role in the physiology, and likely in the pathogenesis, of *Mtb*.

The *Mtb* genome [12] encodes a second protein that is homologous with FGD1 (36% protein sequence identity), annotated as FGD2. Expression of FGD2 (Rv0132c) is under control of the sigma factor SigF [13], which is expressed during stationary growth phase and under stress conditions *in vitro* [14]. A complete genomic microarray analysis revealed that Rv0132c was down–regulated in a *ΔsigF* mutant of *Mtb* in late stationary phase [13]. We set out to determine whether Rv0132c does indeed bind the coenzyme F_420_ and whether it has the annotated F_420_–dependent glucose-6-phosphate dehydrogenase activity. We also noted that Rv0132c has a predicted N–terminal signal sequence that contains motifs suggestive of export via the twin-arginine translocation (Tat) pathway [15], together with post–translational lipid modification.

Here we investigate the possible significance of the Rv0132c protein in F_420_ metabolism in *Mtb*. We report experimental evidence regarding the cellular location of Rv0132c, consistent with post–translational lipidation of the protein. In addition, we show that the Rv0132c protein does bind the cofactor F_420_, has a functional twin–arginine translocation (Tat) signal sequence, and is exported to the cell envelope by the Tat-dependent pathway. These results demonstrate that Rv0132c and the *Mtb* Tat pathway have a direct role in transferring the cofactor F_420_ across the cytoplasmic membrane, and suggest that the known roles of F_420_ should be expanded to include activities in the cell envelope.

## Materials and Methods

Mycobacterial strains and plasmids used in this study are in Table S1, and primers used in the amplification of the various constructs are detailed in Table S2. Full methodological details of bacterial growth, PCR amplification, cloning, homology modeling, western blotting and immunoelectron microscopy are given in the Supporting Information (Text S1).

### Sequence data

Sequence data for Rv0132c and its orthologues in various mycobacterial species were retrieved from the published (www.ncbi.nlm.nih.gov/gene) and unpublished (http://www.sanger.ac.uk/cgi-bin/blast/submitblast/mycobacterium) sequences and the alignments were carried out using CLUSTALW [15].

### Protein expression and purification

The ORF encoding Rv0132c was PCR–amplified from *Mtb* H37Rv genomic DNA (Text S1). When its amino acid sequence is compared with the homologous FGD1, Rv0132c has a 38-residue N-terminal extension, which was predicted by the program PRED–TAT [16] to be a signal sequence with a twin-arginine translocation (Tat) motif. For functional analysis, a construct Rv0132c–Δ38 was therefore prepared that encodes the Rv0132c protein without its predicted signal sequence and with an N–terminal His–tag, cleavable by TEV protease (Text S1). The Rv0132c protein was expressed in *M. smegmatis* mc^2^4517 cells [17], [18], grown for four days, after which the cells were lysed in 20 mM HEPES pH 7.5, 150 mM NaCl, 20 mM imidazole and 1 mM β–ME. After centrifugation, Rv0132c was purified from the supernatant by Ni^2+^–affinity chromatography, cleavage of the His–tag and size exclusion chromatography (SEC) using the same buffer as for lysis (with no imidazole). Analytical SEC was used to determine the oligomeric state of the purified Rv0132c protein, using low molecular weight markers (conalbumin, 75 kDa; ovalbumin, 43 kDa; chymotrypsinogen, 25 kDa; and ribonuclease A, 13.7 kDa; GE Healthcare) to prepare a standard curve (Figure S1). The elution volume of Rv0132c protein was then used to estimate its molecular weight and oligomeric state.

### Rv0132c activity experiments

Assays for the annotated FGD activity of Rv0132c were performed using previously published protocols [5], [6], using appropriate concentrations of the purified Rv0132c, cofactor F_420_ (25 µM) and glucose-6-phosphate (0.01–1 mM). The change in cofactor F_420_ absorbance at 420 nm was monitored over 5 min using UV–visible spectroscopy on a SpectraMAX microplate spectrophotometer (Molecular Devices). The reactions contained 100 µL of 20 mM HEPES pH 7.0, 150 mM NaCl, 1 mM β–ME and were performed in 96–well format (Greiner bio–one, Germany).

### Cofactor characterization and F_420_ preparation

The protein off the gel filtration column was boiled at 100°C for 15 min and centrifuged at 16000×*g*. The resulting supernatant was adjusted to pH<7 with formic acid and applied to a 10 mg C-18 reversed phase solid phase extraction (SPE) cartridge. The SPE cartridge was then washed with 300 µL water and then 150 µL of 50 mM ammonium bicarbonate. The alkaline elution was collected and concentrated to 10 µL and was then diluted to 20 µL with 20% acetonitrile. The resulting solution was back–filled into a static nanoelectrospray needle (tip diameter 4 µm) which was then mounted into a nanoelectrospray interface of a Finnigan LTQ FT mass spectrometer. Ion trap and *ion* cyclotron resonance (ICR) cell data were obtained using a source voltage of 1.3 kV, capillary temperature of 225°C and capillary voltage of 26 V. MS/MS spectra were obtained by isolating key molecular ions and fragmenting using helium as the collision gas and 35% collision energy.

The F_420_ coenzyme was purified from large scale preparations of *M. smegmatis* mc^2^4517 cells expressing *Mtb*–FGD1 or FbiABC (three ORFs involved in the biosynthesis of F_420_) constructs [4] in a 19.5–liter fermentor (New Brunswick Scientific). The purification was carried out using solvent extraction, ion exchange, adsorption and desalting chromatography steps as described before [4], [19]. The absorption of F_420_ at 400 nm (ε_400_ = 25 mM^−1^ cm^−1^) was used to determine its concentration [20].

### Tat–dependent translocation of Rv0132c

The first 42 residues of the Rv0132c protein were used to generate a BlaC fusion construct (Rv0132cSS–'BlaC), which was then transformed into *M. smegmatis* Δ*lys*Δ*blaS* (PM759) [21], *M. smegmatis* Δ*lys*Δ*blaS*Δ*tatA* (JM578) [22] and *M. tuberculosis* Δ*blaC* (PM638) [21] (Text S1). Transformants were tested for carbenicillin resistance by plating on media containing 50 µg/mL carbenicillin, as an indication of Tat-dependent export of 'BlaC fusion constructs. For *M. smegmatis* and *Mtb* strains 500–1000 bacteria were plated on +/− carbenicillin containing agar. Carbenicillin resistance (+) was scored when >90% of colonies plated grew on carbenicillin, whereas carbenicillin sensitivity (−) was defined in strains showing 0% (no single colony growth) on carbenicillin containing media (Text S1).

As an independent method of proving Rv0132c is exported out of the cytoplasm by the Tat pathway, epitope-tagged full-length Rv0132c (Rv0132c-HA) was introduced into *M. smegmatis* mc^2^155 (wild type) [23] and JM576 (*ΔtatC* mutant) [22]. Cells were fractionated after lysis and were then used for western blotting using an anti-HA primary antibody.

### Subcellular localization of native Rv0132c in *Mtb*

The subcellular localization of native Rv0132c in *Mtb* was determined by western blotting and immunoelectron microscopy (Text S1), using polyclonal antibodies raised against purified Rv0132c. The preimmune serum was used as a control. Whole cell lysate (WCL) derived from *Mtb* H37Rv cells was fractionated using differential ultracentrifugation to yield cell wall (CW), cytoplasmic membrane (CM), and soluble (SOL) fractions [24]. In addition, *Mtb* lipoproteins were prepared using Triton X–114 partitioning [24], [25]. For immunogold microscopy, *Mtb* H37Ra cells were fixed and dehydrated in ethanol, pelleted and transferred to fresh resin in gelatin capsules for polymerization overnight at 60°C. Ultrathin sections (∼80 nm) were cut with a 45 degree diamond knife (Diatome) on an EM UC6 and collected onto 400–mesh nickel grids. For immunogold labelling, grids were incubated with either antiserum or preimmune serum, washed, blotted and transferred to drops of secondary antibody (goat anti–rabbit labelled with 10 nm gold, Sigma). Grids were washed, stained, air–dried and viewed in either a Philips CM12 or an FEI Tecnai 12 TEM, both operating at 120 kV.

## Results

### Rv0132c is an F_420_–binding protein

**Figure 2:**
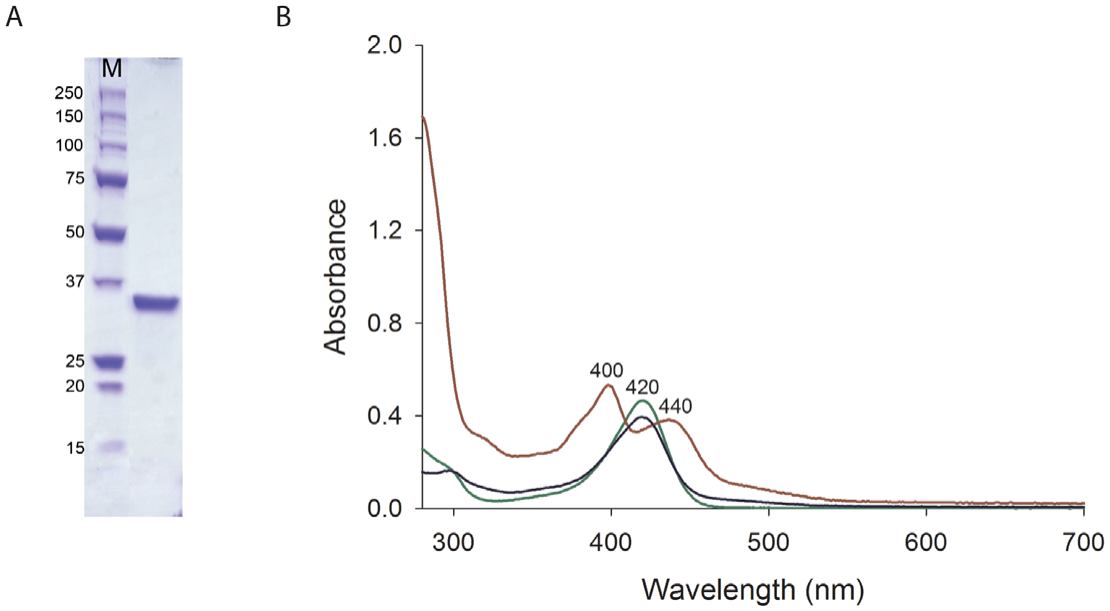
SDS–PAGE and UV–visible spectra of the purified Rv0132c–Δ38 protein. (A) SDS–PAGE gel of the purified Rv0132c–Δ38 protein. (B) UV-visible spectra for purified Rv0132c (0.5 mg/mL in PBS, red), F_420_ extracted from *M. smegmatis* cells (50 µM in PBS, green) and F_420_ extracted from the purified Rv0132c (blue). M: molecular weight markers (kDa).

The Rv0132c protein, expressed in *M. smegmatis* from an Rv0132c–Δ38 construct lacking the predicted N–terminal signal sequence, was purified by Ni–NTA and SEC. The protein eluted in the Ni–NTA step was resolved as a single band on an SDS–PAGE gel (Figure 2A). Analysis of the purified protein by analytical SEC showed that the elution volume corresponded to a molecular weight of 75 kDa (Figure S1), consistent with a dimer in solution when compared with the monomer molecular weight of 35.2 kDa. The purified Rv0132c protein had a light yellow color that was retained after gel filtration, indicating that the source of the color remained bound to the protein.

To characterize the protein–bound yellow color, the purified protein was heat–denatured and the resulting yellow supernatant was analysed by mass spectrometry. This showed that bound F_420_ is indeed responsible for the yellow color of the protein. The mass spectrometry identified F_420_ molecules with varying lengths of poly–glutamate tail, ranging from 4–8 glutamate residues (Figure 1B). This corresponds well with the range of F_420_ species extracted from wild type *M. smegmatis* cells [5], [19] and implies that the protein does not discriminate among F_420_ species with different numbers of glutamate residues. A similar promiscuity of binding has recently been shown for the enzyme Ddn, which binds F_420_-2 and F_420_-5 with similar affinity [26]. These findings are in line with our hypothesis, from studies on FGD1, that the length of the F_420_ poly–glutamate tail may not affect reaction catalysis in F_420_–dependent oxidoreductive enzymes [4].

The absorption spectrum of purified Rv0132c (Figure 2B) shows two absorption peaks, at 400 and 440 nm. In contrast, the F_420_ extracted from the purified Rv0132c shows a single peak at 420 nm, identical to that of the F_420_ extracted from *M. smegmatis* (Figure 2B). This suggests that in the Rv0132c protein environment the F_420_ absorption spectrum is perturbed compared with its free form. Considering that this recombinant Rv0132c–Δ38 lacks its native signal sequence for export and hence is restricted to the cytosol, our findings indicate that Rv0132c binds the F_420_ cofactor in the cytosol, independent of its signal sequence and final destination.

### Rv0132c is incorrectly annotated

**Figure 3:**
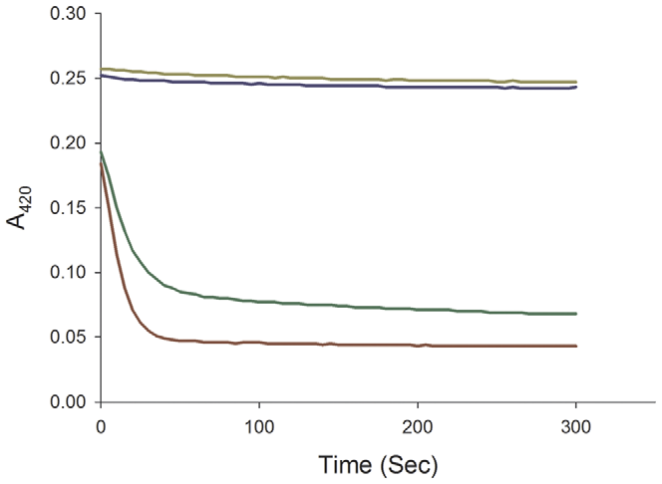
Functional assay of Rv0132c–Δ38 protein. The FGD activity was assessed for Rv0132c–Δ38 protein and *Mtb*–FGD1 as a positive control. *Mtb*–FGD1 shows a decrease at 420 nm absorbance (green and red lines), whereas Rv0132c–Δ38 protein indicated no change in the absorbance (yellow and blue lines). The same results were observed using various concentrations of Rv0132c–Δ38 protein in the presence of different concentrations of glucose-6-phpsphate. The graph shows assays containing 1 µM of each enzyme, 25 µM F_420_ with 0.1 mM (green and yellow lines) and 1 mM (red and blue lines) glucose-6-phosphate.

**Figure 4:**
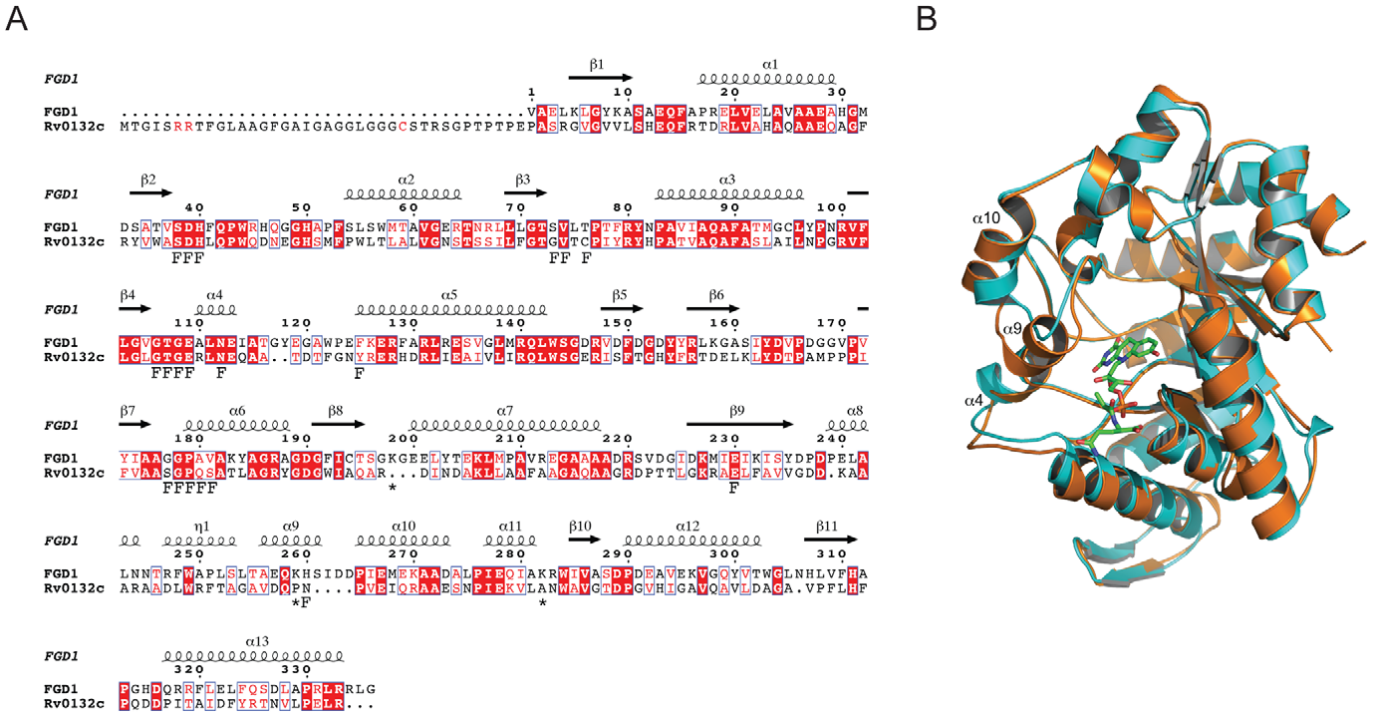
Structural comparison of Rv0132c with FGD1. (A) Amino acid sequence alignment. The secondary structure elements for FGD1 [5] are shown above the sequence. FGD1 residues that hydrogen bond with F_420_ or the phosphate group of glucose-6-phosphate are indicated below the sequence by F and asterisk, respectively. The twin arginines in the Tat motif and the critical cysteine residue in the lipobox motif are shown in red in the Rv0132c signal sequence. (B) Superposition of the FGD1 (orange) crystal structure on the modeled Rv0132c (cyan). The F_420_ cofactor (green) bound to FGD1 is shown in stick representation. Replacement of helix α_9_ with a smaller loop extends the active site cavity in Rv0132c. For details of FGD1 structure see [5].

Of the two *Mtb* gene products annotated with FGD activity, FGD1 has been fully characterized with respect to structure and function [5]. Activity assays over a range of concentrations of enzyme and glucose-6-phosphate failed to detect any FGD activity for Rv0132c, however, under conditions where FGD1 was fully active (Figure 3). This indicates that Rv0132c is mis-annotated as an F_420_-dependent glucose-6-phosphate dehydrogenase. Sequence alignments and homology modeling of Rv0132c (Text S1; Figure 4) show that the F_420_–binding residues identified in FGD1 are also present in Rv0132c but that there are differences in the substrate binding site. In particular, three phosphate binding residues in FGD1, Lys198, Lys259, and Arg283 [5], are not present in Rv0132c. Superposition of the modeled Rv0132c on to the FGD1 experimental structure (Figure 4B) further shows that helix α_9_ in FGD1 is replaced by a smaller loop in Rv0132c, due to a deletion of four residues in this region. This would expand the active site cavity for Rv0132c since this helix is part of the structure that caps the barrel to create the active site cavity [5]. These results suggest that whereas Rv0132c, like FGD1, has the ability to bind F_420_, the two enzymes probably act on different substrates and catalyze different reactions. Our results provide experimental confirmation for the previous bioinformatic prediction that the Rv0132c protein would not have the annotated activity, based on sequence homology [27]. We conclude that *Mtb* possesses just a single enzyme with demonstrated FGD activity (FGD1) and suggest that the suffix 1 should be removed from FGD1 to prevent further confusion with the incorrectly–annotated FGD2.

### Rv0132c and its signal sequence are conserved in pathogenic mycobacteria

Analysis of the Rv0132c sequence (Figure 4A) using the program PRED–TAT [16] predicts a 40-residue N-terminal signal sequence, with a twin-arginine translocation (Tat) motif. The signal sequence also contains a cysteine residue, within a lipobox motif, that implicates Rv0132c as a lipoprotein [27] destined for anchoring to the cell envelope after the post–translational addition of a lipid moiety. Although lipoproteins are mainly translocated across the cytoplasmic membrane using the Sec pathway, it has been reported that some lipoproteins are exported by the Tat system [28], [29], [30], [31], [32].

Sequence searches show that all members of the *Mtb* complex (*M. tuberculosis*, *M. bovis*, *M. africanum*, *M. canetti* and *M. microti*) have homologues of Rv0132c with full conservation (99–100% identity) of the mature protein sequence and the signal sequence, including the Tat motif. Other pathogenic microbacteria such as *M. kansasii*, *M. avium* and *M. avium* subsp. *paratuberculosis* have homologues with lower sequence identity (75–80%) that in most cases retain the Tat export sequence. In contrast, non-pathogenic mycobacteria such as *M. smegmatis* have homologues of Rv0132c that are of much lower sequence identity (30–40%) and all appear to lack a Tat signal sequence.

### Rv0132c possesses a functional Tat signal sequence

The Tat pathway is responsible for transporting folded proteins across the cytoplasmic membrane [33], being different from the Sec pathway, which transports proteins in an unfolded state [34]. Proteins are targeted to the Tat protein translocase, which includes three integral membrane proteins, TatA, TatB and TatC, using an N–terminal signal sequence containing a twin–arginine motif [35]. While the putative Rv0132c signal sequence resembles a Tat export sequence, past studies show that bioinformatic predictions of Tat substrates are problematic and experimental validation of Tat export is critical [36]. Of four tested programs to predict Tat signal sequences, PRED–TAT [16] was the only one to predict Rv0132c protein to be a Tat substrate, emphasizing the need for an experimental approach for verification. To this end we used a BlaC reporter system [22], [36], [37] to determine whether Rv0132c has a functional Tat signal sequence [22]. BlaC of *Mtb* (and BlaS for *M. smegmatis*) is a secreted β–lactamase that confers resistance to β–lactam antibiotics and can be used as a reporter for Tat–dependent export. This is because BlaC is normally exported *via* the Tat pathway [22] and, when expressed without its signal sequence, the truncated BlaC ('BlaC) is not exported, and does not confer β–lactam resistance [22]. In–frame fusion of a functional Tat signal sequence to 'BlaC, however, rescues export and β–lactam resistance [22]. In this way, signal sequences can be tested for their ability to promote Tat–dependent export [22]. Importantly, the 'BlaC reporter only works with Tat (and not Sec) signal sequences.

To determine whether Rv0132c is a Tat substrate, we fused its signal sequence in frame with 'BlaC, forming an Rv0132cSS–'BlaC construct (Text S1). Whereas *M. smegmatis ΔblaS* mutant is sensitive to carbenicillin, a β–lactam antibiotic, expression of Rv0132cSS–'BlaC conferred resistance to carbenicillin, indicating export (Table 1). Similarly, when expressed in *M. tuberculosis ΔblaC*, Rv0132cSS–'BlaC conferred resistance to carbenicillin (Table 1). When expressed in an *M. smegmatis* strain lacking the Tat export channel (Δ*blaS*Δ*tatA*), Rv0132css–'BlaC failed to confer resistance to carbenicillin, confirming Tat dependent export (Table 1). The results obtained for Rv0132cSS–'BlaC were compared with the β–lactam resistance or sensitivity resulting from published controls: full length BlaC, truncated 'BlaC, and a Tat–dependent PlcBss–'BlaC fusion [22]. PlcB is a demonstrated Tat substrate in *Mtb* and PlcBss–'BlaC is Tat exported and confers β–lactam resistance [22], [36]. Rv0132cSS–'BlaC confers a similar level of β–lactam resistance as PlcBss–'BlaC. Taken together, these results demonstrate that Rv0132c has a functional Tat signal sequence.

**Table 1:** Export of an Rv0132cSS–'BlaC^1^ fusion protein is dependent on the Tat pathway.

Strain	Genotype	Carbenicillin Resistance^2^				
		Vector only	BlaC	'BlaC^1^	PlcBss–'Blac	Rv0132cSS–'Blac
*M. smegmatis* PM759	Δ*blaS* 3	−	+	−	+	+
*M. smegmatis* JM578	Δ*blaS*Δ*tatA* 3	−	−	−	−	−
*M. tuberculosis* PM638	Δ*blaC* 4	−	+	−	+	+

1 'BlaC = truncated BlaC lacking its native signal sequence.

2 All strains were resistant to 20 µg/mL kanamycin due to the vector resistance marker. The presence (+) or absence (−) of carbenicillin resistance was determined by colony growth on LB–agar plates plus 20 µg/mL kanamycin and 50 µg/mL carbenicillin for *M. smegmatis* and 7AGT plates plus 20 µg/mL kanamycin and 50 µg/mL carbenicillin for *M. tuberculosis*. See materials and methods for additional experimental details.

3 Carbenicillin resistance was determined after 4–7 days.

4 Carbenicillin resistance was determined after 21 days.

### Rv0132c is exported to the cell envelope by the Tat pathway

**Figure 5:**
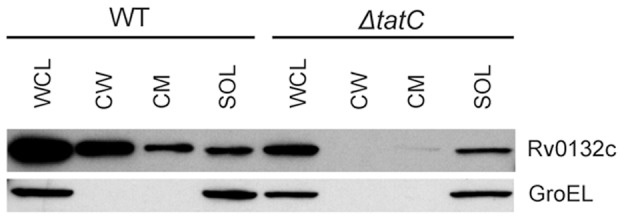
Rv0132c export is Tat dependent. Equalized whole cell lysates (WCL) from wild type (WT) and *ΔtatC M. smegmatis* expressing Rv0132c-HA were fractionated to generate cell wall (CW), cytoplasmic membrane (CM), and soluble (SOL) fractions. Fractions were separated by SDS-PAGE and proteins were detected with an anti-HA antibody. Native GroEL was detected as a cytoplasmic control. Rv0132c-HA was exported to the CW and CM fractions in wild type *M. smegmatis*, but it was not exported in the absence of a functional Tat pathway.

While the 'BlaC reporter experiments demonstrated the existence of a Tat signal sequence in Rv0132c, additional features in the mature domain of a protein are required for it to be Tat exported. This is because the mature domain of Tat-exported proteins must fold prior to export [38], [39]. For this reason, we additionally tested whether export of the full length Rv0132c protein occurs *via* the Tat pathway. Rv0132c with a C-terminal HA epitope tag was expressed in wild type and *ΔtatC M. smegmatis* (Figure 5). Cells were harvested and lysed, and whole cell lysates (WCL) were fractionated using differential ultracentrifugation to generate cell wall (CW), cytoplasmic membrane (CM), and soluble (SOL) fractions (containing the cytosol). To determine cellular localization, equal cell equivalents of the fractions were separated on an SDS-PAGE gel for western blot analysis with anti-HA antibodies (Text S1). Rv0132c was detected as being exported to the cell wall and cell membrane fractions in wild type *M. smegmatis*, but not in the *ΔtatC* mutant where the protein remained in the soluble cytosolic fraction. This analysis demonstrated that Rv0132c export is dependent on a functional Tat pathway. The integrity of the cellular fractions was verified by Western blotting for GroEL (a cytoplasmic protein) as a control. The cell wall and cytoplasmic membrane fractions were free of cytoplasmic contamination, as shown by the lack of GroEL (Figure 5).

### Native Rv0132c is targeted to the cell envelope in *Mtb*

**Figure 6:**
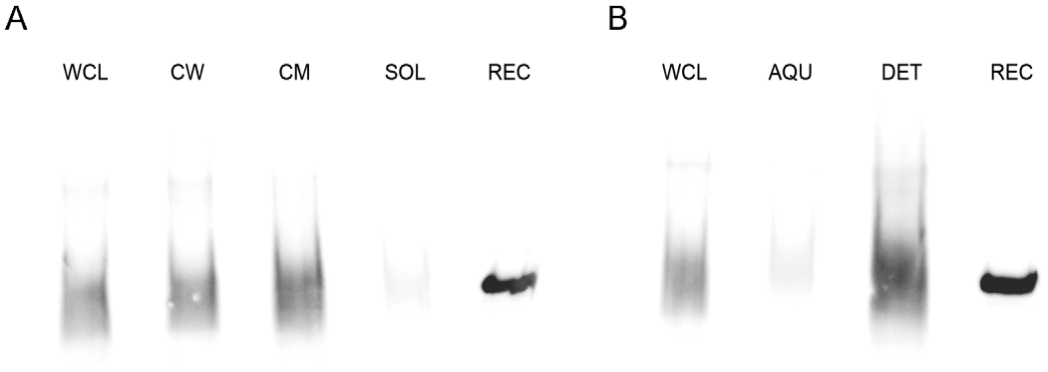
Subcellular localization of Rv0132c protein. (A) Western blots of *M. tuberculosis* H37Rv subcellular fractions using 1/25000 dilution of anti–Rv0132c antiserum. Clear signals are found for the WCL, CW and CM fractions, but not for the SOL fraction. (B) Western blots of Triton X–114 treated fractions. The signal is present only in the DET fraction. WCL: whole cell lysate; CW: cell wall; CM: cytoplasmic membrane; SOL: soluble; AQU: aqueous fraction from Triton X–114 treatment; DET: detergent–enriched fraction from Triton X–114 treatment. In both panels recombinant Rv0132c–Δ38 protein (REC) is used as a positive control (0.7 µg).

To determine the localization of the native Rv0132c protein in *Mtb*, anti–Rv0132c antiserum was used for western blotting and immunoelectron microscopy experiments (Text S1). Whole cell lysate (WCL) prepared from *Mtb* H37Rv cells was fractionated as above to yield cell wall (CW), cytoplasmic membrane (CM), and soluble (SOL) fractions. Equal cell equivalents of the fractions were subjected to western blot analyses, which showed that the native Rv0132c protein is present in the cell wall and membrane fractions of *Mtb* (Figure 6A). Triton X–114 fractionation of *Mtb* H37Rv whole cell lysate further showed that the native Rv0132c protein is present in the detergent–enriched fraction (Figure 6B). Triton X–114 is a non–ionic detergent which has been used extensively to partition hydrophilic proteins (i.e. soluble) from amphiphilic proteins (e.g. lipoproteins and integral membrane proteins) [40]. Given our prior results demonstrating the presence of a functional Tat signal sequence on Rv0132c, the Triton X-114 fractionation results are consistent with Rv0132c being a Tat exported lipoprotein that is exported to the cell envelope. The lipidation of the Rv0132c protein might also explain the reason for smeary bands of native Rv0132c in western blots (Figure 6). In contrast, the recombinant Rv0132c–Δ38 protein used as a control cannot be lipidated as it lacks the signal sequence, and shows a single sharp band. The integrity of the cellular fractions was verified by western blotting using GroEL antibody as a control for cytoplasmic contamination; no GroEL signal was detected in the CW and CM fractions (data not shown).

**Figure 7:**
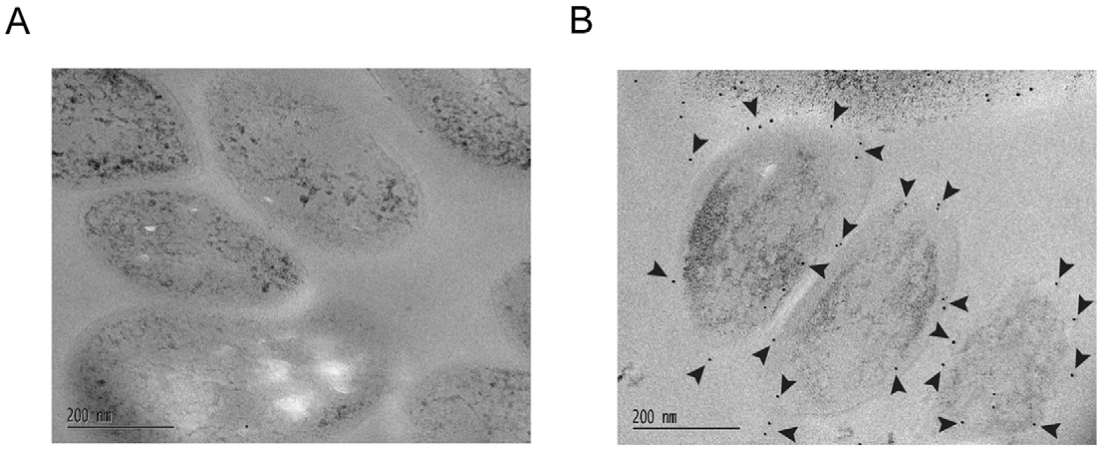
Immunoelectron microscopy of the *M. tuberculosis* H37Ra cells using anti–Rv0132c antiserum. Electron micrographs are shown in which thin cryo–sectioned *Mtb* cells are (A) treated with preimmune serum, and (B) treated with anti-Rv0132c antisera at a dilution of 1/200. The gold particles (indicated by arrowheads) are present mainly on the periphery of the cells in panel B, but are absent from the control panel (A).

More evidence of the Rv0132c protein localization was obtained using immunoelectron microscopy of *Mtb* H37Ra cells using anti–Rv0132c antiserum. The results, which are consistent over a wide range of antiserum concentration, indicate that Rv0132c is indeed present mainly on the periphery of the cells (Figure 7).

## Discussion

The presence of F_420_ in mycobacteria has captured much attention since it was identified as being involved in the activation of a new anti–TB prodrug PA–824 [41]. In a search for enzymes involved in PA–824 activation in *Mtb*, two ORFs were identified, encoding the enzymes FGD1 [41] and Ddn [10]. It was subsequently shown that Ddn directly activates the prodrug using the reduced F_420_ provided by FGD1 [8]. Although the *Mtb* genome has two ORFs encoding proteins annotated as having FGD activity, FGD1 (Rv0407) and FGD2 (Rv0132c) [12], no evidence has been reported for any involvement of Rv0132c in the activation of or resistance towards PA–824. This led us to question the function of Rv0132c and its F_420_–dependent nature; either Rv0132c does not bind F_420_, or it does not have the annotated activity, or is physically separated from Ddn where the activation process occurs.

Our results show unequivocally that Rv0132c is indeed an F_420_-binding protein, but that it does not have the annotated FGD activity. Differences in the substrate-binding region of the active site are consistent with preference for a different, as yet unknown, substrate. What sets Rv0132c apart from FGD1, apart from the differences in the substrate binding site, is its possession (Figure 4A) of a signal sequence containing a twin–arginine translocation (Tat) motif, together with a lipobox motif that mediates lipid modification to produce a cell envelope–anchored lipoprotein. The full–length Rv0132c protein sequence, including its Tat motif, is extremely well conserved (99–100% identity) in the *Mtb* complex (*M. tuberculosis*, *M. bovis*, *M. africanum*, *M. microti* and *M. canetti*). In contrast, although homologues of Rv0132c can be found in non-pathogenic bacterial species they have much lower sequence identity with Rv0132c (<40%) and lack a Tat motif. This suggests a role for Rv0132c and its Tat motif in mycobacterial pathogenesis.

The Tat pathway transports folded proteins across energy–transducing membranes (e.g. the cytoplasmic membrane) [33], being different from the Sec pathway, which transports proteins in an unfolded state [34]. The majority of substrate proteins for the Tat pathway are enzymes that fold in the cytoplasm and require cofactor insertion therein prior to export; examples of the identified cofactors include molydopterin, haem, FAD and NADP^+^ [42]. We have shown here that the signal sequence possessed by Rv0132c, and by implication its homologues in other pathogenic mycobacteria, is functionally active in Tat-mediated export. In addition, using *M. smegmatis* we show Rv0132c is exported in a Tat-dependent manner and we further show the native Rv0132c is exported to the cell envelope of pathogenic *M. tuberculosis*. This further implies translocation of F_420_ to the cell envelope. This is the first report of F_420_ translocation across the cytoplasmic membrane in any F_420_–producing microorganism and expands the number of Tat–dependent cofactors.

Putting our experimental results together, it is reasonable to conclude that the Rv0132c protein binds F_420_ in the cytosol, after which the Rv0132c–F_420_ complex is translocated across the cytoplasmic membrane *via* the Tat pathway, where the protein is anchored to the cell envelope. Although the specific biochemical function of Rv0132c is not yet known, its Tat–dependent translocation may have evolved in pathogenic mycobacteria to enable F_420_ utilization for metabolic or biosynthetic processes in the dense, lipid–rich cell wall. The conservation of Rv0132c and its specific Tat signal sequence in the *Mtb* complex, but not in non–pathogenic mycobacteria implies that the presence of F_420_ outside the cytosol is important to the pathogenesis of tuberculosis. There is growing evidence that F_420_ plays an important part in defense against the host immune system and in non-replicating persistence of *Mtb*. Transition to the persistent state involves major changes in energy metabolism [43], and it has been suggested that F_420_, with its low redox potential, may have a role in reactions associated with anaerobic survival [44].

## Supporting Information

Figure S1 **Calibration curve for estimation of Rv0132c–Δ38 molecular weight using analytical SEC.** The molecular weight calibration curve was obtained by plotting Kav values against LogMW of protein standards. The Kav value determined from the elution volume of Rv0132c–Δ38, indicated with *, corresponded to a molecular weight of 75.4 kDa, which is indicative of a dimer in solution.
(TIF)

Table S1 **Mycobacterial strains and plasmids used in this study.**
(DOCX)

Table S2 **Primers used in the amplification of different Rv0132c constructs.**
(DOCX)

Text S1 **Supplementary methods. Details of bacterial growth; PCR amplification, cloning and preparation of constructs; western blotting; immunoelectron microscopy and homology modeling.**
(DOC)
