# Supplementary figures and images for: Tat–Dependent Translocation of an F420–Binding Protein of Mycobacterium tuberculosis
Source: PLoS One. 2012 Oct 22;7(10):e45003. doi: 10.1371/journal.pone.0045003 (PMC3478262; doi:10.1371/journal.pone.0045003)

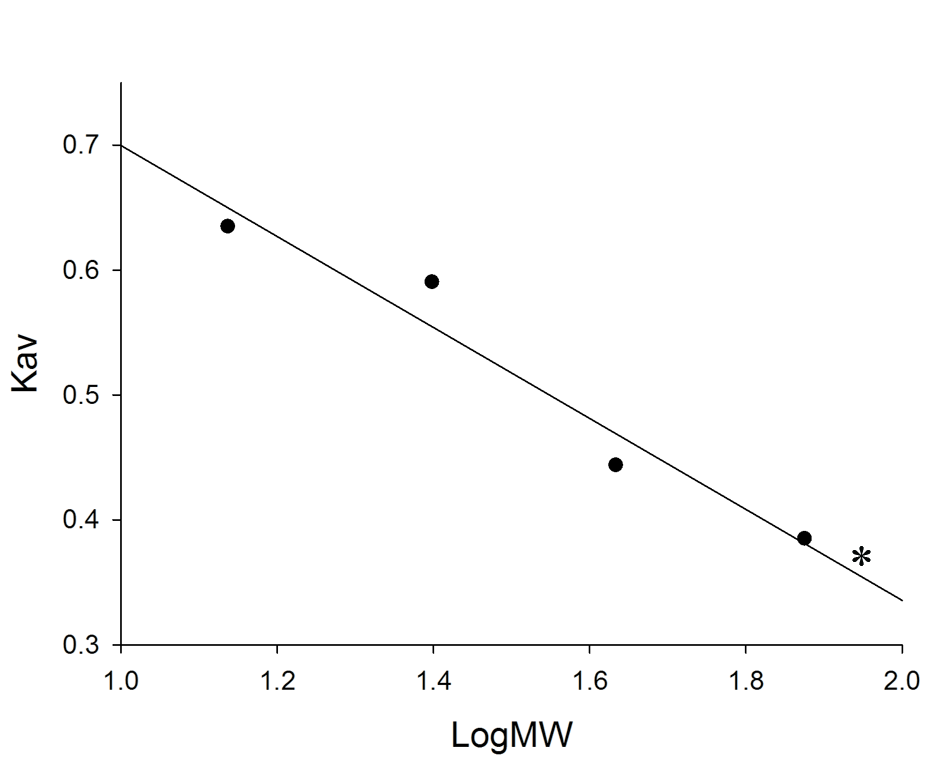

Supplement: Figure S1 — Calibration curve for estimation of Rv0132c–Δ38 molecular weight using analytical SEC. The molecular weight calibration curve was obtained by plotting Kav values against LogMW of protein standards. The Kav value determined from the elution volume of Rv0132c–Δ38, indicated with *, corresponded to a molecular weight of 75.4 kDa, which is indicative of a dimer in solution. (TIF) [file pone.0045003.s001.tif]
