# Supplementary material for: Tat–Dependent Translocation of an F420–Binding Protein of Mycobacterium tuberculosis
Source: PLoS One. 2012 Oct 22;7(10):e45003. doi: 10.1371/journal.pone.0045003 (PMC3478262; doi:10.1371/journal.pone.0045003)
Supplement: Table S1 — Mycobacterial strains and plasmids used in this study. (DOCX) [file pone.0045003.s002.docx]

**Table S1.** Mycobacterial strains and plasmids used in this study

| **Strain** | **Descriptions** | **Ref.** |
| --- | --- | --- |
| PM759 | *Mycobacterium smegmatis ∆lys∆blaS* | [[1](#_ENREF_1)] |
| JM578 | *Mycobacterium smegmatis ∆lys∆blaS∆tatA* | [[3](#_ENREF_3)] |
| JM576 | *Mycobacterium smegmatis* Δ*tatC* | [[3](#_ENREF_3)] |
| mc^2^155 | *Mycobacterium smegmatis wild type* | [[2](#_ENREF_2)] |
| mc^2^4517 | *Mycobacterium smegmatis* mc^2^155 strain harbouring T7 RNA polymerase gene | [[21](#_ENREF_21)] |
| PM638 | *Mycobacterium tuberculosis ∆blaC* | [[1](#_ENREF_1)] |
| H37Rv | *Mycobacterium tuberculosis* | – |
| H37Ra | *Mycobacterium tuberculosis* | – |
| **Plasmid** |  |  |
| pMV261 | Multicopy plasmid with hsp60 promoter, mycobacterial origin of replication and kanamycin resistance cassette | [[8](#_ENREF_8)] |
| pMP327 | BlaC in pMV261 | [[3](#_ENREF_3)] |
| pJM106 | *M. tuberculosis* BlaC mature domain (‘BlaC) in pCC (Epicenter) | [[3](#_ENREF_3)] |
| pJM111 | PlcB signal sequence fused in frame with the mature domain of BlaC in pMV261 | [[3](#_ENREF_3)] |
| pJM113 | The mature domain of BlaC (no signal sequence) in pMV261 | [[3](#_ENREF_3)] |
| pJSC77 | pMV261 with HA tag downstream of hsp60 promoter | [[9](#_ENREF_9)] |
| pEP106 | Cloning intermediate Rv0132c signal sequence in pCR2.1 | This study |
| pEP107 | Cloning intermediate Rv0132c signal sequence in pMV261 | This study |
| pEP108 | Rv0132c signal sequence fused in frame with the mature domain of BlaC | This study |
| pYUB28b | Multicopy shuttle vector with T7 promoter, *E. coli* and mycobacterial origins of replication and hygromycin resistance cassette | [[22](#_ENREF_22)] |
| pDESTsmg | Destination vector for Gateway cloning system, multicopy shuttle vector with T7 promoter, *E. coli* and mycobacterial origins of replication and hygromycin resistance | [[6](#_ENREF_6)] |
| Rv0132c–smg | Rv0132c–Δ38 (no signal sequence) cloned into pDESTsmg | This study |
| Rv0132c-HA | Rv0132c cloned into pJSC77 (HA tag in frame) | This study |
