# Supplementary material for: Tat–Dependent Translocation of an F420–Binding Protein of Mycobacterium tuberculosis
Source: PLoS One. 2012 Oct 22;7(10):e45003. doi: 10.1371/journal.pone.0045003 (PMC3478262; doi:10.1371/journal.pone.0045003)
Supplement: Table S2 — Primers used in the amplification of different Rv0132c constructs. (DOCX) [file pone.0045003.s003.docx]

**Table S2.** Primers used in the amplification of different Rv0132c constructs.

| **Construct** | **Primer Sequences (5’–3’)** | | **Restriction enzyme** |
| --- | --- | --- | --- |
| Rv0132c–smg | Gene–specific  Forward | GGCAGCCGCGCGATGGCAAGCCGCGGGGTG | n/a |
|  | Gene–specific  Reverse | GAAAGCTGGGTGTCAGCGCAGTTCGGGCAGGACGTTGG | n/a |
|  | Generic Forward | GGGGACAAGTTTGTACAAAAAAGCAGGCTTCGAAAACCTGTATTTTCAGGGCAGCGGCGCG | n/a |
|  | Generic Reverse | GGGGACCACTTTGTACAAGAAAGCTGGGTG | n/a |
| Rv0132c–HA | Forward | CAAGCCGCGGGGTGGGTGTCGTCTTATC | n/a |
|  | Reverse | AGGTGCGGTCAAGCTTTGCGCAGTTCGGGCAGGACG | *Hind*III |
| Rv0132cSS–‘BlaC | Forward | TGGCCAGCATGACCGGCATCTCAC | n/a |
|  | Reverse | GGATCCGCGGCTTGCCGGCTCT | n/a |
