# Supplementary material for: Tat–Dependent Translocation of an F420–Binding Protein of Mycobacterium tuberculosis
Source: PLoS One. 2012 Oct 22;7(10):e45003. doi: 10.1371/journal.pone.0045003 (PMC3478262; doi:10.1371/journal.pone.0045003)
Supplement: Text S1 — Supplementary methods. Details of bacterial growth; PCR amplification, cloning and preparation of constructs; western blotting; immunoelectron microscopy and homology modeling. (DOC) [file pone.0045003.s004.doc]

**Text S1. Supplementary methods.**

#### Bacterial growth

*M. tuberculosis ∆blaC* (PM638) (Table S1) was grown in Middlebrook 7H9 or 7H10 (Difco; BD Biosciences) medium supplemented with 0.5% glycerol, 0.05% Tween80 (Fisher) and 1x ADS [0.5% bovine serum albumin, fraction V (Roche), 0.2% dextrose, and 0.85% NaCl]. 7H10 agar was additionally supplemented with 20 µg/mL kanamycin (Acros Chemicals) and 50 µg/mL carbenicillin (Sigma) when necessary. *M. tuberculosis* H37Ra cells were grown in 7H9/ADS/Tween80 for immunoelectron microscopy. *M. smegmatis* wild type (mc^2^155) and *ΔtatC* (JM576) were grown in 7H9 [Middlebrook 7H9 containing 0.5% glycerol, 0.2% glucose, and 0.05% Tween 80].

*M. smegmatis ∆lys∆blaS* (PM759) and *M. smegmatis ∆lys∆blaS∆tatA* (JM578) were grown in Luria–Bertani (LB) medium. LB agar was supplemented with 20 µg/mL kanamycin (Acros Chemicals) and 50 µg/mL carbenicillin (Sigma) when necessary. An *M. smegmatis* mc^2^4517 starter culture was grown at 37 °C in MDG media containing 0.05% Tween80 (25 mM Na_2_HPO_4_, 25 mM KH_2_PO_4_, 50 mM NH_4_Cl, 5 mM Na_2_SO_4_, 2 mM MgSO_4_, 0.5% glucose, 0.25% aspartate, 0.2x metal mix) . The starter culture was grown for 48–72 h at 37 °C and was freshly used at a dilution of 1:100 to inoculate ZYM–5052 autoinduction expression media containing 0.05% Tween80 (1% tryptone, 0.5% yeast extract, 25 mM Na_2_HPO_4_, 25 mM KH_2_PO_4_, 50 mM NH_4_Cl, 5 mM Na_2_SO_4_, 2 mM MgSO_4_, 0.5% glycerol, 0.05% glucose, 0.2% alpha–lactose, 1x metal mix) .

#### PCR amplification and cloning

The ORF encoding Rv0132c was amplified from *M. tuberculosis* H37Rv genomic DNA using PrimeSTAR® HS DNA Polymerase (Takara Bio Inc.) and the primers given in Table S2. The Rv0132c construct without signal sequence was designed by comparison with the sequence of FGD1 (Figure 1), with its start chosen as residue 39 (Δ38). The Rv0132c–Δ38 construct was amplified and cloned using the Gateway® cloning system into the pDESTsmg vector (Table S1) . Briefly, a nested PCR was performed using gene–specific primers in the first round PCR to amplify the Rv0132c–Δ38 construct, after which generic primers were used in the second round of amplification to incorporate the required recombination sites for subsequent cloning. The entry clone was generated by recombination of the PCR products into the pDONR221 vector (Invitrogen) using BP Clonase™ (Invitrogen). The construct was transformed into *E. coli* Top10 cells and plated on LB agar medium containing 50 μg/mL kanamycin. The positive clones were verified using *Bsr*GI digestion and sequencing. The *M. smegmatis* expression construct was produced by recombination of the positive entry clone with pDESTsmg in an LR reaction using LR Clonase™ (Invitrogen). The positive clones were selected on low salt LB agar plates supplemented with 50 μg/mL hygromycin B and were verified using *Bsr*GI digestion.

The signal sequence for Rv0132c protein is predicted as the first 40 residues, using an online Tat signal peptide prediction program, PRED–TAT . To avoid complications from destruction of the cleavage site, the Rv0132c signal sequence fragment used for plasmid construction contained the first 42 residues of Rv0132c. The Rv0132cSS primers (Table S2) were used to amplify the first 42 amino acids of Rv0132c from *M. tuberculosis* H37Rv genomic DNA. The fragment was inserted into pCR2.1 (Invitrogen) to form pEP106. The pEP106 and pMV261 vectors were digested with *Msc*I and *BamH*I and ligated to form pEP107. The BlaC mature domain fragment (‘BlaC) was digested from pJM106 with *Bgl*II and ligated with pEP107 (digested with *BamH*I) to form the final construct, pEP108, with the Rv0132c signal sequence fused in frame of ‘BlaC (Rv0132cSS–‘BlaC). The Rv0132cSS–‘BlaC construct was then transformed into *M. smegmatis ∆lys∆blaS* (PM759) *, M. smegmatis ∆lys∆blaS∆tatA* (JM578) and *M. tuberculosis ∆blaC* (PM638) . Transformants were tested for carbenicillin resistance by plating on media containing 50 µg/mL carbenicillin.

For the translocation experiments in *M. smegmatis*, the full-length Rv0132c open reading frame was amplified from *M. tuberculosis* genomic DNA using the primers outlined in Table S2. The pJSC77 vector was double-digested with *Msc*I and *Hind*III restriction enzymes and the *Hind*III-digested Rv0132c fragment was then ligated into the vector using T4 DNA ligase (Roche Applied Science). The stop codon at the end of the Rv0132c open reading frame was deleted, to allow in-frame translation of HA tag at the C-terminus. The positive clones were selected on LB agar plates containing 50 μg/mL kanamycin and were subsequently verified using restriction digestion and sequencing.

#### Western blotting

An *M. tuberculosis* H37Rv culture was inoculated at OD_600_=0.04 into 200 mL 7AGT (Middlebrook 7H9/ADS/Tween80 media containing 0.5% glycerol). The cells were harvested at OD_600_=1.16 on day five by centrifugation. The cell pellets were then sterilized by gamma–irradiation for removal from the BSL–3. The cells were subsequently resuspended in 5 mL lysis buffer [PBS, 0.6 mg/ml each of DNase and RNase, and a cocktail of protease inhibitors (2 mg/mL each of aprotinin, E–64, leupeptin, and pepstatin A and 100 mg/mL Pefabloc SC)]. 4 mL were lysed by five passages in a French pressure cell at 20,000 psi and unbroken cells were pelleted at 3,000 ×g for 30 min to generate clarified whole cell lysates (WCLs, 7.4 mg/mL). 3 mL of WCL was fractionated into cell wall, cytoplasmic membrane, and soluble components using differential ultracentrifugation . Briefly, WCL was centrifuged at 50,000 ×g (30 min) to pellet the cell wall fraction. The resulting supernatant was centrifuged at 100,000 ×g (2 hours) to separate the cell membrane fraction (pellet) from the soluble fraction (supernatant). The cell wall and cytoplasmic membrane fractions were washed once with the lysis buffer, re–centrifuged and subsequently re–suspended in 1.5 mL of the lysis buffer. All centrifugation steps were performed at 4°C. The protein concentrations in different fractions were determined using the BCA method and BSA as a standard.

*M. tuberculosis* H37Rv lipoproteins were prepared using Triton X–114 partitioning . The whole cell lysate from the previous section was incubated with Triton X–114 at a final concentration of 2%, overnight at 4°C. The insoluble material was subsequently removed by centrifugation at 3000 ×g for 10 min at 4°C. The detergent and aqueous phases were separated by raising the temperature to 37°C followed by centrifugation at 3000 ×g for 10 min at room temperature. Both aqueous (upper) and detergent (lower) phases were re–extracted once to prevent cross contamination. Proteins from the detergent phase were precipitated using acetone (10 volumes at –20 °C) after which the proteins were resuspended in the lysis buffer. The protein concentration in both fractions was determined as described earlier.

*M. smegmatis* cultures were grown in 7H9 media to OD_600_ 0.5-1.0. Cells were lysed and fractionated as described for H37Rv. Fractions were run on 1D-PAGE and transferred to nitrocellulose membrane (Whatman). Rv0132c-HA was detected using an anti-HA mouse primary antibody (Covance). Native GroEL was detected using an anti-His mouse primary antibody (Abgent). The anti-His antibody recognizes a string of endogenous histidines in *M. smegmatis* GroEL . The secondary antibody was goat anti-mouse conjugated to HRP (Bio-Rad). Signal was detected using Western Lighting ECL (Perkin-Elmer).

#### Immunoelectron microscopy

*M. tuberculosis* H37Ra cells were prepared for immunogold analysis by fixing in 4% paraformaldehyde in 0.1 M phosphate buffer, performed overnight on a rotating platform at 4 ºC. Fixed bacteria were dehydrated in an ethanol series (30, 50, 70, 90 and 100%; 15–20 min each with gentle agitation). Minimal centrifugation was used to pellet the bacteria for ethanol changes, and pellets were resuspended by vortexing. For resin infiltration, samples were incubated in 2:1 (by volume) ethanol:LR White resin (medium grade, London Resin) for 1 hour, then 1:2 ethanol: LR White for a further hour, and neat LR White for 48 hours with several changes. Pellets were then transferred to fresh resin in gelatin capsules and polymerized overnight at 60 ºC. Ultrathin sections (~80 nm) were cut with a 45 degree diamond knife (Diatome) on an EM UC6 ultramicrotome (Leica Microsystems) and collected onto 400–mesh nickel grids.

For immunogold labelling, grids were incubated on 50 µL drops of blocking buffer [10% (v/v) normal goat serum, 4% (w/v) BSA, 10% (w/v) cold water fish skin gelatin in PBS] for 20 min at room temperature (RT), then on drops of either antiserum or preimmune serum diluted 1:4000 in rinse buffer [1%(v/v) normal goat serum in PBS] for 5 h at RT or overnight at 4 ºC. Grids were then washed on 6× 50 µL drops of rinse buffer (5 min each with gentle agitation), blotted and transferred to drops of secondary antibody (goat anti–rabbit labelled with 10 nm gold, Sigma) diluted 1:200 in rinse buffer, for 2 h at RT. Grids were then washed 6× on rinse buffer followed by 2× on dH_2_O (5 min each with gentle agitation), then stained with 2% (w/v) aqueous uranyl acetate (20 min at RT), washed 6× on dH_2_O (30 s each), then stained with Reynolds’ lead citrate (3 min at RT) and washed 6× on dH_2_O (30 s each). Grids were air–dried and viewed in either a Philips CM12 or an FEI Tecnai 12 TEM, both operating at 120 kV. Images were recorded on either a Gatan Bioscan CCD camera or a Gatan Ultrascan 1000 CCD camera.

**Homology modeling**

A sequence comparison between Rv0132c and FGD1 (Figure 1) shows that the two proteins share 36% protein sequence identity, implying extremely similar three–dimensional structures, and reveals that the F_420_–binding residues identified in FGD1 are also present in Rv0132c. There are, however, differences in the substrate binding site. Three phosphate binding residues in FGD1, Lys198, Lys259, and Arg283 , are not present in Rv0132c (Figure 3A), suggesting that whereas Rv0132c, like FGD1, has the ability to bind F_420_, the two enzymes may act on different substrates and catalyze different reactions.

Since no experimental three-dimensional structure was available for the Rv0132c protein, homology–based modeling with MODELLER (<http://salilab.org/modeller/>). was used to obtain a three–dimensional model for Rv0132c, based on the experimental three–dimensional structure of FGD1 . .MODELLER performs comparative protein structure modeling by satisfaction of spatial restraints . A sequence alignment of Rv0132c with that of FGD1 was carried out using CLUSTALW [16] and MODELLER automatically calculated a model containing all non–hydrogen atoms. The resulting model was visualised and compared to the FGD1 experimental structure using COOT .

Superposition of the modelled Rv0132c on to the FGD1 experimental structure (Figure 1) revealed that helix α_9_ in FGD1 is replaced by a smaller loop in Rv0132c, due to a deletion of four residues in this region. This would expand the active site cavity for Rv0132c. This helix is part of the sequence motif IS3 in FGD1, which is involved in capping the barrel to create the active site cavity . In FGD1, it also contains the residue Lys259, which is involved in binding to the phosphate group of glucose-6-phosphate . This further supports the conclusion, from the sequence alignment results, that Rv0132c may be an F_420_–dependent enzyme with a different, but unknown, substrate.

#### References

1. Flores AR, Parsons LM, Pavelka MS, Jr. (2005) Genetic analysis of the beta-lactamases of Mycobacterium tuberculosis and Mycobacterium smegmatis and susceptibility to beta-lactam antibiotics. Microbiology 151: 521-532.

2. Snapper SB, Melton RE, Mustafa S, Kieser T, Jacobs JWR (1990) Isolation and characterization of efficient plasmid transformation mutants of *Mycobacterium smegmatis*. Mol Microbiol 4: 1911-1919.

3. McDonough JA, Hacker KE, Flores AR, Pavelka MS, Jr., Braunstein M (2005) The twin-arginine translocation pathway of Mycobacterium smegmatis is functional and required for the export of mycobacterial beta-lactamases. J Bacteriol 187: 7667-7679.

4. Bashiri G, Squire CJ, Baker EN, Moreland NJ (2007) Expression, purification and crystallization of native and selenomethionine labeled *Mycobacterium tuberculosis* FGD1 (Rv0407) using a *Mycobacterium smegmatis* expression system. Protein Expr Purif 54: 38-44.

5. Studier FW (2005) Protein production by auto-induction in high-density shaking cultures. Protein Expr Purif 41: 207-234.

6. Goldstone RM, Moreland NJ, Bashiri G, Baker EN, Shaun Lott J (2008) A new Gateway vector and expression protocol for fast and efficient recombinant protein expression in Mycobacterium smegmatis. Protein Expr Purif 57: 81-87.

7. Bagos PG, Nikolaou EP, Liakopoulos TD, Tsirigos KD (2010) Combined prediction of Tat and Sec signal peptides with hidden Markov models. Bioinformatics 26: 2811-2817.

8. Stover CK, de la Cruz VF, Fuerst TR, Burlein JE, Benson LA, et al. (1991) New use of BCG for recombinant vaccines. Nature 351: 456-460.

9. Glickman MS, Cox JS, Jacobs WR, Jr. (2000) A novel mycolic acid cyclopropane synthetase is required for cording, persistence, and virulence of Mycobacterium tuberculosis. Mol Cell 5: 717-727.

10. Gibbons HS, Wolschendorf F, Abshire M, Niederweis M, Braunstein M (2007) Identification of two Mycobacterium smegmatis lipoproteins exported by a SecA2-dependent pathway. J Bacteriol 189: 5090-5100.

11. D'Orazio M, Folcarelli S, Mariani F, Colizzi V, Rotilio G, et al. (2001) Lipid modification of the Cu,Zn superoxide dismutase from Mycobacterium tuberculosis. Biochem J 359: 17-22.

12. Rengarajan J, Murphy E, Park A, Krone CL, Hett EC, et al. (2008) Mycobacterium tuberculosis Rv2224c modulates innate immune responses. Proc Natl Acad Sci USA 105: 264-269.

13. Bashiri G, Squire CJ, Moreland NJ, Baker EN (2008) Crystal structures of F420-dependent glucose-6-phosphate dehydrogenase FGD1 involved in the activation of the anti-tuberculosis drug candidate PA-824 reveal the basis of coenzyme and substrate binding. J Biol Chem 283: 17531-17541.

14. Sali A, Blundell TL (1993) Comparative protein modelling by satisfaction of spatial restraints. J Mol Biol 234: 779-815.

15. Emsley P, Cowtan K (2004) Coot: Model-Building Tools for Molecular Graphics. Acta Crystallogr D Biol Crystallogr 60,: 2126-2132.

16. Wang F, Jain P, Gulten G, Liu Z, Feng Y, et al. (2010) Mycobacterium tuberculosis dihydrofolate reductase is not a target relevant to the antitubercular activity of isoniazid. Antimicrob Agents Chemother 54: 3776-3782.

17. Bashiri G, Rehan AM, Greenwood DR, Dickson JMJ, Baker EN (2010) Metabolic Engineering of Cofactor F420 Production in Mycobacterium smegmatis. PLoS ONE 5: e15803.
